# Recent Advances of the Zebrafish Model in the Discovery of Marine Bioactive Molecules

**DOI:** 10.3390/md22120540

**Published:** 2024-11-30

**Authors:** Changyu Liu, Jiaxun Li, Dexu Wang, Jibin Liu, Kechun Liu, Peihai Li, Yun Zhang

**Affiliations:** 1 Biology Institute, Qilu University of Technology (Shandong Academy of Sciences), Jinan 250103, China; liuchangyu000422@163.com (C.L.); 18287319631@163.com (J.L.); dexuwangbio@163.com (D.W.); liujibin310@163.com (J.L.); hliukch@sdas.org (K.L.); 2 Engineering Research Center of Zebrafish Models for Human Diseases and Drug Screening of Shandong Province, Key Laboratory for Biosensor of Shandong Province, Jinan 250103, China

**Keywords:** zebrafish model, marine natural products, bioactivity evaluation

## Abstract

Marine natural products are increasingly utilized in nutrition, cosmetics, and medicine, garnering significant attention from researchers globally. With the expansion of marine resource exploration in recent years, the demand for marine natural products has risen, necessitating rapid and cost-effective activity evaluations using model organisms. Zebrafish, a valuable vertebrate model, has become an efficient tool for screening and identifying safe, active molecules from marine natural products. This review, based on nearly 10 years of literature, summarizes the current status and progress of zebrafish models in evaluating marine natural product bioactivity. It also highlights their potential in exploring marine resources with health benefits, offering a reference for the future development and utilization of marine biological resources.

## 1. Introduction

Covering over 70% of the Earth’s surface, the ocean is the cradle of life and the largest known habitat. The marine biosphere hosts a vast diversity of species and immense biomass, comprising over 80% of the Earth’s total biomass, making it a significant reservoir of natural resources [1,2]. Compared to terrestrial environments, marine conditions—such as high salinity, pressure, oligotrophy, darkness, and extreme temperatures—have driven marine organisms to develop unique metabolic pathways and defense mechanisms. These adaptations enable the production of biologically active substances with complex and diverse structures, offering valuable opportunities to discover novel marine-derived compounds with bioactive potential [3,4]. In recent years, numerous marine compounds with antitumor, anti-inflammatory, antioxidant, and cardiovascular protective properties have been identified. These compounds hold significant promise for use in nutrition, cosmetics, pharmaceuticals, and other biotechnology sectors closely linked to human health [5]. Consequently, marine natural products have drawn extensive attention from researchers worldwide. In medicine, marine natural products are a major source of modern drugs [6]. Currently, 20 marine-derived drugs have received market approval, with dozens more in clinical trials, playing pivotal roles in treating cardiovascular diseases, cancer, and infections [7].

Bioactivity screening is a crucial step in drug discovery. Traditional in vitro activity screening, based on cells and enzymes, has evolved to offer high-throughput, rapid, sensitive, and cost-effective evaluations, thanks to advances in automation and instrumentation [8]. However, in vitro screening has its limitations. For example, in vitro cultured cells cannot replicate the extracellular microenvironment or disease progression. Many specialized cell types and organs cannot be cultured in vitro [9]. In contrast, in vivo bioactivity assays based on animal models provide more biologically relevant insights. Bioactive natural products identified in vivo are more likely to show activity in mammalian models [10]. Common animal models include invertebrates (e.g., nematode, fruit fly), non-mammalian vertebrates (e.g., zebrafish), and mammals (e.g., mouse). Among these, zebrafish (*Danio rerio*) has emerged as an excellent model, bridging flies/nematodes and mice/humans for studying embryonic development and gene function [8,11].

**Figure 1:**
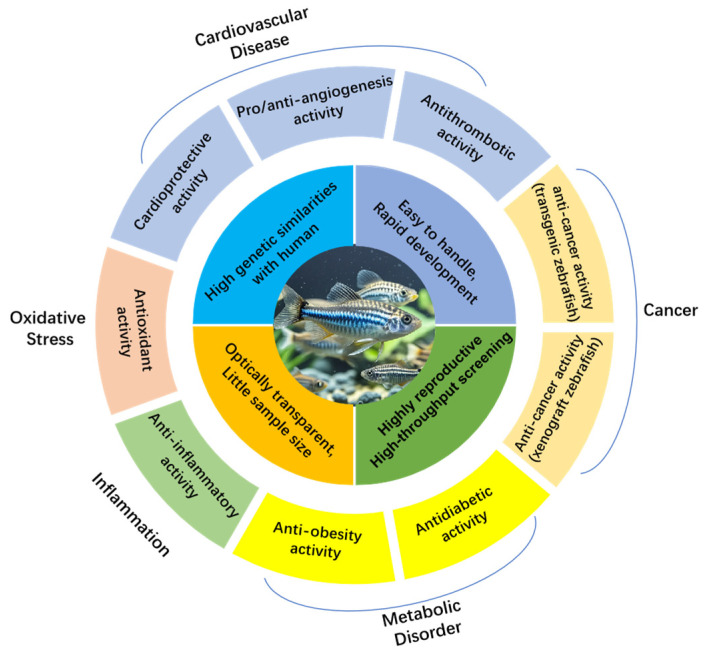
Application of zebrafish models in bioactivity evaluations of marine natural products.

First introduced as an experimental model in the 1950s [12,13], zebrafish embryos were later used to study genetics, bringing zebrafish to the forefront of experimental biology [14,15]. The use of zebrafish for chemical screening in microplates began in 2000 [16]. Since then, zebrafish have become a powerful model for rapidly analyzing gene function and bioactivity. Several reviews have discussed zebrafish’s role in screening for antitumor, cardiovascular, and neuroprotective natural products, as well as safety evaluations [8,17,18,19]. However, there remains a gap in reviews specifically addressing the use of zebrafish models in evaluating marine natural product bioactivity. This article aims to fill this gap by summarizing the application of zebrafish in screening marine bioactive natural products. It covers zebrafish biology and key studies involving zebrafish in discovering marine bioactive molecules for nearly 10 years. The bioactivities discussed in this article include cardioprotection, proangiogenesis, antiangiogenesis, antithrombosis, anticancer, antidiabetes, anti-obesity, antioxidation, and anti-inflammation (Figure 1). This review seeks to raise awareness of zebrafish models, expand their application, and promote the development of marine natural products.

## 2. Zebrafish as a Model Species

Zebrafish are small tropical freshwater fish belonging to the genus *Danio* in the family Cyprinidae. They originate from South Asia, including countries such as Bangladesh and India. Adult zebrafish are 3–5 cm in length, with a slender, spindle-shaped body featuring blue and silver stripes on their surface [20,21]. Zebrafish are easy to breed due to their gentle temperament and tolerance to various environmental conditions. Optimal laboratory breeding conditions include a temperature of 28 °C, pH levels of 7–8, a 14:10 h light–dark cycle, and salinity of 0.25–0.6 ppm [17,22]. Zebrafish embryos are small, measuring 1–5 mm, and develop rapidly, reaching adulthood within approximately three months. The reproductive cycle is about one week, and a female can lay 200–300 eggs in one spawning [9].

Zebrafish are ideal for studying tissue and organ development due to their rapid development and short sexual maturity cycle. They are highly reproductive, providing an abundant supply of embryos for experiments. Embryos and larvae are small, require minimal amounts of test samples, and are easy to handle in microplates [23]. The embryos are optically transparent, allowing in vivo observation of organ and tissue development without damage. The simple administration of test samples, which can be diluted in sterile water or dimethyl sulfoxide, enables quick absorption through the zebrafish’s skin and gills [8,24]. Zebrafish can generate large numbers of synchronously developing transparent embryos, ensuring that all relevant tissues and organs form within a few days post-fertilization. This minimizes the sample amount needed for in vivo experiments at microgram levels, enabling high-throughput phenotypic screening on microplates, which is essential for discovering bioactive natural products [25,26]. The zebrafish model offers high reproducibility, providing reliable statistical data for validation and allowing rapid in vivo bioactivity analysis at microgram levels, even with limited sample sizes [8]. Determining biological activity in zebrafish offers advantages such as high sensitivity, stable internal testing, and the ability to detect chemicals with similar modes of action or sites of activity [27].

Zebrafish share significant physiological and genetic similarities with mammals. They have more than 70% genome homology with humans and up to 82% homology with human disease-related genes. Zebrafish also possess genes associated with the cell cycle, growth, differentiation, tissue function, tumorigenesis, and suppression [28]. Compared to fruit flies and nematodes, zebrafish represent a more complete vertebrate system, with physiological and pharmacological responses similar to those of humans [29]. These characteristics make zebrafish highly suitable for identifying drugs and bioactive natural products with therapeutic potential [30]. Additionally, zebrafish provide economic and experimental advantages over mammalian models, reducing the number of mammals used in research, cutting maintenance costs, shortening experimental cycles, and facilitating high-throughput pharmacological screening—all in adherence to the 3R principles (Replacement, Reduction, and Refinement) [22,31,32]. Therefore, zebrafish offer distinct advantages over fruit flies, nematodes, and mammals in biological and biomedical research (Table 1). Their whole biological complexity combined with high-throughput screening capabilities makes zebrafish an ideal model for screening marine bioactive natural products, which have low production and are difficult to obtain.

Since the mid-1990s, large-scale genetic screenings have produced hundreds of zebrafish mutant lines with various disease-related phenotypes [10]. Following the completion of zebrafish genome sequencing, research using zebrafish models has progressed rapidly. Techniques such as gene editing and transgenesis have facilitated the construction of various transgenic zebrafish lines with specific fluorescent protein expression in cells, tissues, and organs. These models have been successfully applied to pathological studies and drug screening [9,33,34,35,36]. For example, transgenic zebrafish *Tg(fli1: EGFP)* are used for screening vascular promoters and inhibitors, *Tg(CD41: EGFP)* for antithrombotic drugs and *Tg(cmlc2: GFP)* for cardioprotective drugs. Casper mutant zebrafish are commonly employed in cancer and stem cell research [37,38,39,40,41]. Zebrafish models also play a vital role in toxicology research, elucidating the functions of specific genes and their interactions with signaling pathways. The Wellcome Trust Sanger Institute in the UK has established over 3000 mutant zebrafish lines related to human diseases through large-scale mutagenesis screening [42].

## 3. Zebrafish Models for Evaluating Marine Bioactive Natural Products

Choosing an appropriate high-throughput screening model is essential for identifying lead compounds in drug discovery. With innovations in zebrafish disease models and advancements in screening technologies, zebrafish have become increasingly valuable for screening marine bioactive natural products. Various zebrafish models have been employed to efficiently explore marine bioactive substances. Below, we highlight the progress of zebrafish models in the investigation of marine bioactive compounds (Table 2), particularly regarding cardiovascular diseases, cancers, metabolic diseases, inflammation, and oxidative stress over the past decade.

### 3.1. Zebrafish Models Related to Cardiovascular Diseases

Cardiovascular diseases encompass various conditions affecting the heart and vascular system, with complex etiologies involving environmental and genetic factors. These diseases pose significant health risks, emphasizing the need for safe, effective cardiovascular drugs. Zebrafish exhibit strong similarities to humans in terms of cardiac action potentials, electrocardiograms, vascular structures, and genes governing heart development. The conservation of cardiovascular drug responses between zebrafish and humans underpins zebrafish’s role in cardiovascular drug discovery and toxicology research [43,44]. Zebrafish embryos’ transparency and rapid cardiovascular development enable non-invasive in vivo imaging of heart function. Structural and functional changes, including heart chambers, heartbeat, blood flow, and vasculature, are easily observed. Transgenic zebrafish expressing fluorescent proteins in endothelial, cardiac, or red blood cells are frequently used to study cardiovascular and hematopoietic system development [45]. Here, we review the use of zebrafish models to discover marine natural products with cardioprotective, antithrombotic, and vascular regulatory properties.

#### 3.1.1. Evaluation of Cardioprotective Activity by Zebrafish Models

Cardiac function is crucial for diagnosing and treating cardiovascular diseases. The zebrafish heart, composed of the sinus venosus, atrium, ventricle, and bulbus arteriosus, is located in the anterior abdomen between the pleura and thorax [46]. By 48 h post-fertilization, zebrafish embryos have typically completed heart development and begin circulating blood through contraction and relaxation [47]. Therefore, zebrafish at this stage are commonly used to evaluate cardioprotective activity. Key indicators of cardiac function include heart morphology, heart rate, sinus venosus–bulbus arteriosus (SV–BA) distance, stroke volume, and ejection fraction [48]. Gene editing techniques have produced zebrafish strains expressing fluorescent proteins in the heart, such as *Tg(cmlc2:EGFP)*, *Tg(myl7:EGFP)* and *Tg(-5.1myl7:DsRed2-NLS)* [48]. Using these strains, models of heart failure, arrhythmias, and other cardiac diseases have been established using drugs like verapamil, doxorubicin, terfenadine, and aconitine. These models facilitate the screening and identification of bioactive molecules with cardioprotective effects.

Currently, zebrafish cardioprotective models have been successfully established and applied for screening marine molecules with anti-heart failure and anti-arrhythmic effects. For example, using the AB or *Tg(cmlc2:EGFP)* zebrafish strain, our group developed cardioprotective models with verapamil and terfenadine. We identified phospholipids from shrimp heads, squid gonads, and viscera, such as phosphatidylcholine and phosphatidylethanolamine, which demonstrated anti-heart failure and anti-arrhythmic effects [49,50,51,52]. Additionally, a peptide PcShK3 from the zoantharian *Palythoa caribaeorum* exhibited significant cardioprotective activity at low concentrations [53]. Pott et al. discovered okadaic acid and calyculin A from sponges, using ILK-deficient msq mutant zebrafish embryos, which showed anti-heart failure activity [54]. Zebrafish-based heart failure and arrhythmia models provide a valuable platform for identifying cardioprotective marine molecules, supporting their further development and application.

#### 3.1.2. Evaluation of Angiogenesis Regulatory Activity by Zebrafish Models

Angiogenesis, the formation of new capillary networks, involves endothelial cell proliferation and migration. In zebrafish, fluorescent reporter genes driven by vascular-specific promoters, such as *flk1:EGFP* or *fli1:EGFP*, allow direct visualization of angiogenesis [55]. Angiogenesis primarily occurs via sprouting, regulated by vascular endothelial growth factor (VEGF) [56]. VEGF-A, secreted by somatic cells, is crucial for inducing intersegmental vessel growth in the dorsal aorta and serves as a target for angiogenic therapies [57,58]. Angiogenesis, regulated by proangiogenic and inhibitory factors, is critical for maintaining vascular balance. Disruption of this balance may result in excessive or reduced angiogenesis, contributing to various diseases [56].

Inadequate angiogenesis can lead to ischemic cardiovascular diseases, such as myocardial infarction, angina pectoris, and coronary heart disease [59]. Developing angiogenesis-promoting drugs may aid in treating these conditions, and marine natural products offer potential sources. Zebrafish models have identified several marine compounds with proangiogenic activity. Using *Tg(fli-1:EGFP)* or *Tg(vegfr2:GFP)* zebrafish lines, our team established an intersegmental vessel defect model with PTK787, discovering many compounds with proangiogenic activity like dinotoamide J [60], communesin I, fumiquinazoline Q, and protuboxepin E [61], N-(2-hydroxyphenyl)-acetamide, ethyl formyltyrosinate [62], chaetofanixins A–E [63], bialorastin C [64], sterigmatocystin A [65], agelanemoechine [66], lemnardosinanes A [67], marchaetoglobin B and C [68], chevalinulins A and B [69], clavukoellian K [70], chaetoviridin L, chaetomugilin A, and chaephilone D [71]. Additionally, marine compounds like fucoidan LMWF [72], ZoaNPY [73], Cyclotripeptide X-13 [59], and Pestaphilone J [74], as summarized in Table 2.

Diseases linked to excessive angiogenesis, including tumors, retinopathies, rheumatoid arthritis, and moyamoya disease, require anti-angiogenic therapies [75]. Currently, anti-angiogenic research primarily focuses on inhibiting tumor angiogenesis, which plays a pivotal role in tumor growth, invasion, and metastasis and is recognized as one of cancer’s hallmarks [76]. Several anti-angiogenic drugs have been approved for cancer treatment. Numerous anti-angiogenic compounds have been identified from marine sources using zebrafish models (Table 1), including pyrrolidinedione AD0157 [76], catunaregin [77], bis(2,3-dibromo-4,5-dihydroxybenzyl) ether (BDDE) [78], stellettin B [79], Ishophloroglucin A [80], polypeptide CS5931 [81], Diphlorethohydroxycarmalol [82], protein ASP-3 [83], polysaccharide SPS [84], quinadoline B [85], phloroglucinol and dieckol [86], Sinularin [87], capnellene GB9 [88], Toluquinol [89], Solomonamide A [90], Somocystinamide A [91], Fucoidan [92], dihydroaustrasulfone alcohol WA-25 [93], bis(2,3,6-tribromo-4,5-dihydroxybenzyl) ether (BTDE) [94], asperhiratide [95], pyrrole-pyridinimidazole derivative 8a [96], penisterine C and D [97], monacolin X [98], Dolastatin 15 [99], and murrangatin [100].

#### 3.1.3. Evaluation of Antithrombotic Activity by Zebrafish Models

Thrombosis is a serious cardiovascular condition posing a significant threat to human life. Genes related to coagulation, anticoagulation, and platelet signaling in zebrafish show high homology with those in humans. Studies have shown that internal and external coagulation factors, as well as multiple platelet surface receptors found in human blood and platelets, also exist in zebrafish [101]. Zebrafish possess cellular and humoral hemostatic mechanisms similar to those in humans, making them an excellent model for studying thrombosis and antithrombotic drugs. Zebrafish exhibit similar responses to commonly used clinical anticoagulants and antiplatelet drugs, thus mimicking human hemostasis and thrombosis [102,103]. The transparency of zebrafish embryos allows dynamic observation of arterial or venous thrombosis formation under a microscope, and zebrafish thrombus models are stable and reproducible, making them a valuable tool for antithrombotic research.

Blood clot formation can result in myocardial infarction, ischemia, and stroke, which are major global causes of death. Developing effective, safe therapies for thrombotic diseases is a significant challenge [104]. Zebrafish thrombosis models can be induced by phenylhydrazine (PHZ) [105], ferric chloride [106,107], arachidonic acid [108], microinjection, or direct immersion. These models have been instrumental in identifying safe, effective antithrombotic natural products. Antithrombotic efficacy is typically assessed by staining wild-type zebrafish larvae with o-dianisidine and evaluating the red blood cell staining intensity in the heart or observing venous thrombosis morphology under a fluorescence microscope. Additionally, transgenic zebrafish strains such as *Tg(CD41:EGFP)* and *Tg(LCR:EGFP)*, which express green fluorescent protein in platelets and red blood cells, offer models for dynamically evaluating antithrombotic activity [109].

Various marine natural products with antithrombotic activity have been identified (Table 2). For example, using the arachidonic acid-induced thrombosis model of the AB zebrafish strain, our group discovered that 4-hydroxyphenylacetic acid [110], sarcoeleganolide I [111], sarcoelegan C [112], and sarcocinerenolides C and H [113], derived from marine fungi and soft corals, as well as phospholipids from shrimp heads, squid viscera, and gonads [49,50,51,52], exhibit antithrombotic effects. Additionally, Yang et al. identified a pyruvylated and sulfated galactan (PSG) with antithrombotic activity from the green alga *Dictyosphaeria cavernosa* using the phenylhydrazine-induced thrombosis model in *Tg(gata1:dSRed)* and *Tg(CD41:EGFP)* zebrafish strains [114].

### 3.2. Zebrafish Models Related to Cancer

Zebrafish have become essential animal models for human tumor research, aiding in the preclinical development of anticancer drugs. Various zebrafish models for cancer research have been developed, categorized into induced, transgenic, and implanted models [17,115]. Among these, transgenic and implanted zebrafish tumor models have been applied to marine natural product research.

#### 3.2.1. Evaluation of Anticancer Activity by Transgenic Zebrafish Models

Transgenic zebrafish models are developed through gene knockdown or gene editing in the whole organism or specific organs [116]. Zebrafish share significant homology with human cancer-related genes, making gene manipulation feasible. These models have rapidly become valuable tools for cancer research [17,117]. One model involves inducing tumors by regulating the abnormal expression of oncogenes or tumor suppressor genes. For instance, a T-lymphocyte leukemia model is created by overexpressing the mouse oncogene *c-myc* under the zebrafish lymphocyte gene promoter *rag2* [118]. Additionally, by knocking out the tumor suppressor gene *Rb1* in a *mitf-BRaf* and *p53 −/−* background, highly aggressive melanoma cells are induced, creating an immune-compatible melanoma zebrafish model [119]. Compounds like Oligo-Fucoidan [120] and Terphenyllin Derivative CHNQD-00824 [121] have shown antitumor activity using this model. Though many such models are available, few have been used to evaluate the anticancer potential of marine natural products.

Another important model for cancer research involves inhibiting angiogenesis, a process critical for tumor growth and metastasis. As effective angiogenesis inhibitors are key in cancer treatment [122], these zebrafish models of antiangiogenesis activity are commonly used to screen for anticancer compounds from marine sources. Reviews have summarized transgenic zebrafish models used in cancer research, highlighting key developments, advantages, and limitations [117,122,123,124].

#### 3.2.2. Evaluation of Anticancer Activity by Xenograft Zebrafish Models

Implanted zebrafish tumor models, primarily xenograft models, involve implanting fluorescent protein-labeled tumor cells or tissues into zebrafish embryos or adults. This allows real-time observation of cancer cell proliferation and metastasis and is a widely used method with a high success rate [116]. The optimal time for tumor cell implantation is within 48 h post-fertilization. Implantation sites include the yolk sac, perivitelline space, and duct of Cuvier, as well as the abdominal cavity in adult zebrafish [115,125]. Since the first zebrafish xenograft model was introduced in 2005, the technique has matured and is widely used in different cancer types [123]. These models use commercial human cancer cell lines or primary tumor cells from cancers such as melanoma, breast, colorectal, pancreatic, ovarian, renal, lung, and oral cancers [126]. Researchers can control tumor cell implantation in different zebrafish locations to meet specific research goals. Numerous marine antitumor molecules have been discovered using these models (Table 2), including rakicidin B and B1 [127], rhopaloic acid A [128,129], tilapia piscidin 4 [130], crambescidine-816 [131], holothurian glycosaminoglycan [132], saringosterol acetate [133], cyclo(l-Pro-l-Leu), cyclo(l-Pro-l-Val), cyclo(l-Pro-l-Phe), cyclo(l-Pro-l-Tyr) [134], intestinal peptide SCIP [135], anticancer peptides MP06 [136], isofistularin-3 [137], and trabectedin [138].

### 3.3. Zebrafish Models Related to Metabolic Disorder

Metabolic disorders like diabetes and obesity, along with related conditions like hyperglycemia, hyperlipidemia, hypertension, and fatty liver disease, are emerging as major public health concerns [139,140,141]. Zebrafish models have proven useful for studying these conditions and exploring treatments. Their advantages make them an ideal model for screening marine natural products that may prevent or treat metabolic disorders [142,143].

#### 3.3.1. Evaluation of Antidiabetic Activity by Zebrafish Models

Diabetes is a condition characterized by chronic hyperglycemia [144]. The morphogenesis and basic cellular structure of the zebrafish pancreas shares similarities with mammals, consisting of an exocrine gland (ductal, acinar, and centroacinar cells) and an endocrine gland (α-cells, β-cells, δ-cells, ε-cells, and pp-cells) [145]. Hormones like glucagon, insulin, and somatostatin regulate glucose levels [146,147]. The development and signaling pathways involved in the zebrafish endocrine pancreas are highly homologous to mammals, and the exocrine gland is morphologically and ultrastructurally conserved, making zebrafish a suitable model for diabetes research [142]. Normal blood glucose levels in zebrafish (50–75 mg/dL) are comparable to humans (70–120 mg/dL) [148].

Diabetes is mainly categorized into type 1 and type 2. Type 1 diabetes is a chronic autoimmune destruction characterized by the loss of insulin-producing β-cells in the pancreas, leading to insulin deficiency, and often occurs in children [149]. Type 2 diabetes is primarily due to acquired insulin resistance, which usually occurs along with decreased insulin secretion, accounts for 90% of all diabetes cases, and is often associated with obesity [150,151]. Diabetic zebrafish models are created using chemical induction, glucose immersion, transgenic techniques, or gene knockout, resulting in hyperglycemia and insulin resistance [152,153]. Several antidiabetic components have been identified from marine natural products using zebrafish models (Table 2), including brasilterpenes A and C [154], penipyrol C [155], *Polysiphonia japonica* extracts and 5-bromoprotocatechualdehyde [156,157], Con-Ins peptides [158], aspterric acid [159], Antarctic krill enzymatic hydrolysates [160], and dieckol [161].

#### 3.3.2. Evaluation of Anti-Obesity Activity by Zebrafish Models

Obesity is a complex metabolic disorder characterized by excessive lipid accumulation in the body and significant increase in free fatty acids and triglycerides in the serum and often caused by high-fat, high-sugar diets and lack of exercise [17]. It increases the risk of chronic diseases such as diabetes, cardiovascular disease, and cancer [141]. The lipid metabolism networks and the hypothalamic circuits regulating energy balance in zebrafish are highly conserved with humans [162,163,164]. Zebrafish store excess nutrients in white adipocytes as lipid droplets, which can be visualized using neutral dyes like Oil Red O or Nile Red, facilitating the study of hypolipidemic effects [165,166]. The similarities in lipid storage and metabolism, along with zebrafish’s advantages (short life cycle, ease of use, and low drug dosage), make them ideal for obesity research and anti-obesity drug screening.

Obese zebrafish models can be generated via diet-induced obesity or transgenic technology. Diet-induced models are primarily used for lipid-lowering screenings, while genetically engineered models provide insight into obesity mechanisms [167]. Using these models, several marine compounds with anti-obesity and lipid-lowering effects have been discovered (Table 2). For example, Ralph Urbatzka’s team conducted a zebrafish Nile red fat metabolism assay by a diet-induced obese zebrafish model and found that extracts from seagrass, blue-green algae, and marine actinomycetes, along with compounds like 13-2-hydroxypheophytine, vitamin K1-analog (OH-PhQ), citreorosein, questinol, (1′Z)-2-(1′,5′-dimethylhexa-1′,4′-dieny1)-5-methylbenzene-1,4-diol, 6-(3-hydroxy-6-methyl-1,5-heptadien-2-yl)-3-methylbenzene-1,4-diol, 4-hydroxy-3,7-dimethyl-7-(3-methylbut-2-en-1-yl)benzofuran-15-one, 1,8-epoxy-1(6),2,4,7,10-bisaborapentaen-4-ol, and 9-(3,3-dimethyloxiran-2-yl)-1,7-dimethyl-7-chromen-4-ol, exhibited anti-obesity activities [168,169,170,171,172,173,174,175,176,177]. Additionally, studies have shown that red seaweed Palmaria mollis [178], polysaccharide SS3-N1 [179], fucoxanthin [180], glycosaminoglycans [181], and saringosterol acetate [182] exhibit promising anti-obesity effects.

### 3.4. Zebrafish Models Related to Inflammation

Inflammation is a series of defensive physiological responses to internal and external stimuli, involving multiple cytokines that regulate the inflammatory system’s balance [183]. Excessive or prolonged inflammation can cause the immune system to damage normal tissues, impair functions, and trigger pathological reactions [184]. Diseases like cardiovascular disease, cancer, diabetes, and Alzheimer’s are linked to inflammation [185,186]. The zebrafish have highly conserved natural and acquired immune systems, and its immune cell types, immune signal transduction and functions are similar to those of mammals [187]. Zebrafish natural immune systems can quickly respond to infections and tissue damage, easily causing inflammatory reactions, making them ideal for studying inflammation and wound repair [188]. The development of transgenic zebrafish strains with fluorescent immune cell markers, along with the transparency of zebrafish embryos, allows for real-time tracking of immune responses [189].

The triggering factors of inflammation include mechanical damage, chemical induction, bacterial and viral infections, etc. At present, zebrafish experiments mainly use three methods to simulate inflammation of the immune system: local inflammation induced by tail cutting, systemic inflammation induced by lipopolysaccharide, and acute inflammation induced by copper sulfate. Several transgenic zebrafish strains have been developed, such as *Tg (lysC: EGFP)*, *Tg (lysC: DsRED2)*, and *Tg (coro1a: EGFP)*, to label neutrophils and macrophages [186]. The zebrafish inflammation model is widely used to discover marine bioactive molecules (Table 2). Our team used *Tg (zlyz: EGFP)* zebrafish to identify anti-inflammatory compounds, including peptide LLTRAGL [190], Septosone A [191], Somallactam A [192], Dysidinoid B [193], Sarcoelegan variants [112], Altechromone A [194], Purpurols A and B [195], and protein hydrolysate AJH-1 [196]. Other teams have identified molecules like apo-9′-fucoxanthinone [197], 5-hydroxypalisadin B [198], fucoidan SFF-PS-F5 [199], fucoidan LJSF4 [200], fucose containing sulfated polysaccharides FCSP [201], 24-Methylcholesta-5, 22-Diene-3β-ol [202], sulfated polysaccharides CFCE-PS [203], sulfated polysaccharides SFPS [204], sulfated Galactofucan LJNF3 [205], maxol and dieckol [206], enzymatic peptide SEP [207], and Micrometam C [208].

### 3.5. Zebrafish Models Related to Oxidative Stress

Oxidative stress occurs when the body’s oxidation or antioxidant systems become imbalanced, leading to excessive production of reactive oxygen species (ROS) [209]. When the original dynamic balance of redox is disrupted, a large amount of oxidative intermediate ROS are generated and cause cell structural changes and genetic damage [210]. Prolonged oxidative stress can result in pathological conditions, including cardiovascular disease, inflammation, cancer, and metabolic disorders [211]. Zebrafish genes involved in oxidative processes are highly conserved, and many proteins share functions with their mammalian counterparts [212]. Zebrafish have become a valuable model organism for studying antioxidant mechanisms and screening marine-derived antioxidant compounds in vivo.

The construction of zebrafish oxidative stress models mainly involves two methods: the physical hypoxia method and chemical drug intervention. The physical hypoxia method uses nitrogen perfusion and Gaspak anaerobic techniques, while the chemical drug intervention method utilizes compounds such as AAPH, sodium sulfite, and H2O2 to induce oxidative stress [209]. After oxidative induction, indicators like ROS, nitric oxide, antioxidant enzyme activity, malondialdehyde, heart rate, and mortality rate are measured to evaluate the antioxidant capacity of natural products. Zebrafish oxidative stress models have been widely applied in discovering marine bioactive molecules (Table 2). For instance, using metronidazole-induced *Tg (krt4: NTR hKikGR)^cy17^* zebrafish, our team identified peptides YSQLENEFDR and YIAEDEAR with antioxidant activity from sea snails [213], and Frondoplysin A from the marine sponge *Dysidia fructosa* [214]. Other antioxidant components, such as peptide AFFP [215], seahorse peptide SHP [216], phenoglucinol, eckol, dieckol, and trihalomethaneol A [217], agaro-oligosaccharides AO [218], xyloketal B [219], (−)-loliolide [220], protein hydrolysate HPH [221], and fucoidan HFPS-F4 [222], have also been discovered.

**Table 2:** Application of zebrafish models in the bioactivity evaluation of marine natural products.

Marine Nature Products	Source	Models	Main Effects (Concentrations)	References
Phospholipids	Shrimp heads, squid gonads, and viscera	AB or *Tg(cmlc2: EGFP)* zebrafish strain	Anti-heart failure and anti-arrhythmic effects (25, 50, 100 μg/mL and 20, 40, 80 μg/mL)	[49,50,51,52]
Peptide PcShK3	zoantharian *Palythoa caribaeorum*	*Tg(cmlc2: GFP)* zebrafish strain	Cardio-protective activity (20 μM)	[53]
Calyculin A and okadaic acid	sponges	ILK-deficient msq mutant zebrafish embryos	Anti-heart failure activity (100 and 0.15 μM)	[54]
Dinotoamide J	Marine-derived fungus *Aspergillus austroafricanus* Y32-2	*Tg (vegfr2: GFP)* zebrafish model	Proangiogenic activity (70, 120 μg/mL)	[60]
Communesin I, Fumiquinazoline Q and Protuboxepin E	Marine-derived fungus *Penicillium expansum* Y32	*Tg (vegfr2: GFP)* zebrafish model	Proangiogenic activity (20 and 50 and 100 μg/mL)	[61]
N-(2-hydroxyphenyl)-acetamide, ethyl formyltyrosinate	Marine-derived fungus *Penicillium chrysogenum* Y20-2	*Tg (FLI1: EGFP)* zebrafish model	Proangiogenic activity (25, 50, 100 μg/mL)	[62]
Chaetofanixins A-E	Hadal trench-derived fungus *Chaetomium globosum* YP-106	*Tg (flk1: EGFP)* zebrafish model	Proangiogenic activity (20, 40, 80 μg/mL)	[63]
Bialorastin C	Deep-sea cold-seep-derived fungus *Penicillium bialowiezense* CS-283	*Tg(vegfr2: GFP)* zebrafish model	Proangiogenic activity (20, 40 μM)	[64]
Sterigmatocystin A	Sponge-derived fungus *Aspergillus versicolor* (15XS43ZD-1)	*Tg (vegfr2: GFP)* zebrafish model	promoting angiogenesis activity (1.25 μM)	[65]
Agelanemoechine	South China sea sponge *Agelas nemoechinata*	*Tg (vegfr2: GFP)* zebrafish model	Proangiogenic activity (5 μM)	[66]
Lemnardosinanes A	Soft coral *Lemnalia* sp.	*Tg (vegfr2: GFP)* zebrafish model	Proangiogenic activity (20 μM)	[67]
Marchaetoglobin B and C	Marine-sponge-associated fungus *Chaetomium globosum* 162105	*Tg (vegfr2: GFP)* zebrafish model	Proangiogenic activity (80 μM)	[68]
Chevalinulins A and B	Deep-sea cold-seep-derived fungus *Aspergillus chevalieri* CS-122	*Tg (vegfr2: GFP)* zebrafish model	Proangiogenic activity (40 and 80 μg/mL)	[69]
Clavukoellian K	Marine soft coral *Lemnalia* sp	*Tg (vegfr2: GFP)* zebrafish model	Proangiogenic activity (2.5 μM)	[70]
Chaetoviridin L, chaetomugilin A, and chaephilone D	Hadal trench-derived fungus *Chaetomium globosum* YP-106	*Tg (flk1: EGFP)* zebrafish model	Proangiogenic activity (20, 40, 80 μg/mL)	[71]
Fucoidan LMWF	Brown algae *Saccharina japonica*	High-glucose-induced zebrafish with blood vessel growth inhibition	Promotes subintestinal vessel formation in angiogenesis (100 μg/mL)	[72]
Polypeptide ZoaNPY	*Zoanthus sociatus*	*Tg (Fli1a: EGFP)* zebrafish model	Proangiogenic effects (100 pmol)	[73]
Cyclotripeptide X-13	Mangrove fungus *Xylaria* sp. (no. 2508)	*Tg (fli1: EGFP)* zebrafish model	Proangiogenic activity (10, 50, 100 μM)	[59]
Pestaphilone J	Sea-mud-derived fungus *Neopestalotiopsis* sp. HN-1-6	*Tg (flk1:EGFP)* zebrafish model	Proangiogenic activity (40 μM)	[74]
Pyrrolidinedione AD0157	Marine fungi	*Tg (fli1:EGFP)y1* zebrafish model	Anti-angiogenic activity (10 µM)	[76]
Catunaregin	Stem bark of *Catunaregam spinosa*	*Tg (fli1: EGFP)* zebrafish model	Anti-angiogenic activity (10, 50, 100 μM)	[77]
Bis(2,3-dibromo-4,5-dihydroxybenzyl) ether (BDDE)	Marine algae Leathesia nana and *Rhodomela confervoides*	Zebrafish with Alkaline phosphatase staining	Anti-angiogenic activity (6.25, 12.5, 25 μM)	[78]
Stellettin B	Marine-sponge *Stelletta* sp.	*Tg (fli1: EGFP)y1* transgenic zebrafish	Anti-angiogenic activity (≥50 nM)	[79]
Ishophloroglucin A	*Ishige okamurae*	*Tg (flk: EGFP)* zebrafish with high glucose-induced angiogenesis	Anti-angiogenic activity (0.015, 0.05, 0.15, 0.5 µM)	[80]
Polypeptide CS5931	*Ciona savignyi*	Zebrafish model	Anti-angiogenic Activity (10, 20, 30 μg/mL)	[81]
Diphlorethohydroxycarmalol	brown alga *Ishige okamurae*	*Tg (flk: EGFP)* zebrafish with high glucose-induced angiogenesis	Anti-angiogenic Activity (0.06, 0.2, 0.6, 2 μM)	[82]
Protein ASP-3	*Arca subcrenata Lischke*	*Tg (fli1: GFP)* zebrafish model	Anti-angiogenic Activity(18.8–150 μg/mL)	[83]
Polysaccharide SPS	Brown seaweed *Sargassum integerrimum*	*Tg (fli1a:EGFP)y1* zebrafish model	Anti-angiogenic Activity (0, 1, 4 mg/mL)	[84]
Quinadoline B	marine-derived fungus *Aspergillus clavutus* LZD32-24	*Tg (fli1a: EGFP)* zebrafish model	Anti-angiogenic Activity (2, 5, 10 μM)	[85]
Phloroglucinol and dieckol	Brown alga *Ecklonia cava*	*Tg (flk: EGFP)* zebrafish under high glucose conditions	Anti-angiogenic activity (0.24, 0.8, 2.4, 8 and 0.03, 0.1, 0.3, 1 μM)	[86]
Sinularin	Soft coral *Sinularia flexibilis*	The angiofluorescent zebrafish	Anti-angiogenic activity (5 μM)	[87]
Capnellene GB9	Soft coral *Capnella imbricata*	*Tg (fli: EGFP)* zebrafish model	Anti-angiogenic activity (10 μM)	[88]
Toluquinol	Marine fungus *Penicillium* sp. HL-85-ALS5- R004	*Tg (fli1: EGFP)y1* zebrafish model	Anti-angiogenic activity (20 μM)	[89]
Solomonamide A	Marine algae *Leathesia nana*	Zebrafish model	Anti-angiogenic activity (5, 10 μM)	[90]
Somocystinamide A	Marine microorganisms *Lyngbya majuscula*	*Tg(fli1: EGFP)* zebrafish model	Anti-angiogenic activity (80, 160, 300, 1.6, 3 μM)	[91]
Fucoidan	*Fucus vesiculosus*	*Tg(fli1: EGFP)* zebrafish model	Anti-angiogenic activity (300 μg/mL)	[92]
Dihydroaustrasulfone alcohol WA-25	Soft coral *Cladiella australis*	*Tg(fli1: EGFP)y1* and *Tg(kdrl: mCherryci5-fli1a: negfpy7)* zebrafish model	Anti-angiogenic activity (50 μM)	[93]
Bis(2,3,6-tribromo-4,5-dihydroxybenzyl)ether (BTDE)	Marine red alga *Symphyocladia latiuscula*	*Tg (flk1: EGFP)* zebrafish model	Anti-angiogenic activity (2.5–10 μM)	[94]
Asperhiratide	Soft coral-derived fungus *Aspergillus hiratsukae* SCSIO 5Bn1003	*Tg (fli1: EGFP)* zebrafish model	Anti-angiogenic activity	[95]
Pyrrole-pyridinimidazole derivative 8a	Marine sponge *Agelas nakamurai*	*Tg (flk1: EGFP)* zebrafish model	Anti-angiogenic activity(100, 150 μM)	[96]
Penisterine C and D	Marine brown alga derived fungus, *Penicillium* *sumatraense* SC29	*Tg (fli1: EGFP)* zebrafish model	Anti-angiogenic activity (10.2, 20.4 and 8.6, 17.2 μg/mL)	[97]
Monacolin X	Fungi-NMK7 associated with marine sponge	*Tg (Kdr: EGFP)/ko1* zebrafish model	Anti-angiogenic activity (0.5, 1 μM)	[98]
Dolastatin 15	Marine cyanobacteria	*vhl*+/*hu2117* heterozygous parents carrying the *fli1a:egfp^y1^* transgene	Anti-vascularization effect (6 μM)	[99]
Murrangatin	Marine plant	*Tg (fli1: EGFP)* zebrafish model	Anti-angiogenic effects (10, 50, 100 μM)	[100]
4-hydroxyphenylacetic acid	Marine-derived fungus *Emericellopsis maritima* Y39–2	Arachidonic Acid induced AB zebrafish model	Antithrombotic activity(164.3, 328.6 μM)	[110]
Sarcoeleganolide I	Soft coral *Sarcophyton elegans*	Arachidonic Acid induced AB zebrafish model	Antithrombotic activity (20 μM)	[111]
Sarcoelegan C	Soft coral *Sarcophyton elegans*	Arachidonic Acid induced AB zebrafish model	Antithrombotic activity (20 μM)	[112]
sarcocinerenolides C and H	soft coral *Sarcophyton cinereum*	Arachidonic Acid induced AB zebrafish model	Antithrombotic activity (20 μM)	[113]
Pyruvylated and sulfated galactan (PSG)	Green alga *Dictyosphaeria cavernosa*	phenylhydrazine-induced thrombosis model of *Tg(gata1: dSRed)* and *Tg(CD41: EGFP)* zebrafish strains	Antithrombotic activity (100, 150 μg/mL)	[114]
Oligo-Fucoidan	Brown seaweed	AB, *Tg (fabp10a: HBV-HBx-mCherry, myl7: EGFP)*, *Tg (fabp10a: src, myl7: EGFP*), *Tg (fabp10a: HBV-HBx-mCherry, myl7: EGFP, p53−/+)*, *Tg (fabp10a: src, myl7: EGFP, p53−/+)*	Prevents radiation-induced fibrosis and secondary tumors (300 mg/kg)	[120]
Terphenyllin derivative CHNQD-00824	Marine-derived compound library	*Tg (fabp10: rtTA2s-M2; TRE2: EGFP krasG12V)*	inhibit DOX-induced liver-specific enlargement (2.5, 5 μM)	[121]
Rakicidin B and B1	Marine *Micromonospora*	Zebrafish xenotransplantation model with HCT-8 tumor cell	Antitumor (3, 10,30, 35, 40 ng/mL)	[127]
Rhopaloic acid A	Marine sponge *Rhopaloeides* sp.	Zebrafish oral and Bladder cancer xenotransplantation model	Antitumor effects against oral and Bladder cancer (0.03, 0.3 μg/mL)	[128,129]
Tilapia piscidin 4	*Nile tilapia*	AB zebrafish bladder cancer model	Antitumor effects against bladder cancer (0.3, 1, 3 μg/mL)	[130]
Crambescidine-816	Marine sponge *Crambe crambe*	Zebrafish xenotransplantation model with colorectal carcinoma cells	antitumor activity against colorectal carcinoma (1, 5, 10 μM)	[131]
Holothurian glycosaminoglycan	Sea cucumber *Holothuria leucospilota*	AB/Tubingen zebrafish xenotransplantation model with B16F10 tumor cell	Antitumor Effects (1 μM)	[132]
Saringosterol acetate	Brown alga *Hizikia fusiforme*	*Tg(fli1: EGFP)* zebrafish hepatocellular carcinoma xenograft model	Suppress hepatocellular carcinoma growth and metastasis (2 or 5 μg/g)	[133]
Cyclo (l-Pro-l-Leu), cyclo (l-Pro-l-Val), cyclo (l-Pro-l-Phe) and cyclo (l-Pro-l-Tyr)	*Exiguobacterium* *acetylicum* S01	zebrafish xenogroft model with HT-29 tumor cells	Inhibit the tumor progression (50, 100, 150 μM)	[134]
Intestinal peptide (SCIP)	Sea cucumber	AB zebrafish xenogroft model with MCF-7 tumor cells	Inhibits the proliferation of MCF-7 tumor cells (27.8, 83.3, 250 μg/mL)	[135]
Peptides MP06	Green sea algae *Bryopsis plumosa*	*Tg(kdrl: GFP)* zebrafish xenogroft model with A549 cells	Reduce metastatic dissemination (1, 2, 4, 10 μM)	[136]
Isofistularin-3	Marine sponge *Aplysina aerophoba*	zebrafish xenogroft model with VampiroPC3 or Vampiro-SH-SY5Y cells	Antiproliferative activity (5, 10, 25, 50 μM)	[137]
Trabectedin	Marine derived *Ecteinascidia turbinata*	zebrafish xenogroft model with NCI-H295R, MUC-1, and TVBF-7 cells	Reduce ACC cell xenograft area and metastasis formation (15 nM)	[138]
Brasilterpenes A and C	Deep Sea-Derived Fungus *Paraconiothyrium brasiliense* HDN15-135	*Tg(-1.2ins:htBidTE*–*ON; LR)* zebrafish model	Hypoglycemic activity (0, 1, 10, 50, 200 μM)	[154]
Penipyrol C	Mangrove derived fungus *Penicillium* sp. HDN-11-131	*Tg(-1.2ins: H2BmCherry) and Tg (-1.2ins: H2BmCherry)* zebrafish model	Anti-diabetes (10 μM)	[155]
Extracts of Polysiphonia japonica and 5-Bromoprotocatechualdehyde	*Polysiphonia japonica*	*Tg(ins: EGFP)* zebrafish model	Protects against palmitate-induced β-cell dysfunction (10 and 50 μM)	[156,157]
Con-Ins G1, Con-Ins G3, Con-Ins T1A, Con-Ins T2, Con-Ins K1, Con-Ins K2	*Conus geographus*, *C.tulipa*, *C.kinoshitai*	Streptozotocin-induced model of diabetes in zebrafish	Reduce blood glucose (65 ng/g)	[158]
Aspterric acid	Mangrove sediment-derived fungus *Penicillium polonicum* H175	*Tg (Ins: htBidTEON; LR)* zebrafish model	Hypoglycaemic effect (10 μmol/L)	[159]
Antarctic krill enzymatic hydrolysates AKEH	Antarctic krill	Diet-induced diabetic zebrafish model	Hypoglycaemic effect (1.35,2.70, 5.40 g/L)	[160]
Dieckol	*Ecklonia cava*	Alloxan-induced diabetic zebrafish model	Anti-diabetes activity (1 µg/g body weight)	[161]
*Palmaria mollis*	The red seaweed *Palmaria mollis*	Diet-induced obese zebrafish model	Anti-obesity effects (PM dose of 2.5% (*w*/*w*))	[178]
Citreorosein and questinol	Marine sponge-associated fungus *Talaromyces stipitatus* KUFA 0207.	Diet-induced obese zebrafish model	Anti-obesity effects (5 μM)	[176]
(1′Z)-2-(1′,5′-dimethylhexa-1′,4′-dieny1)-5-methylbenzene-1,4-diol, 6-(3-hydroxy-6-methyl-1,5-heptadien-2-yl)-3-methylbenzene-1,4-diol, 4-hydroxy-3,7-dimethyl-7-(3-methylbut-2-en-1-yl)benzofuran-15-one, 1,8-epoxy-1(6),2,4,7,10-bisaborapentaen-4-ol, 9-(3,3-dimethyloxiran-2-yl)-1,7-dimethyl-7-chromen-4-ol	Marine sponge *Myrmekioderma* sp.	Diet-induced obese zebrafish model	Lipid-reducing activity (10 µM)	[177]
Polysaccharide SS3-N1	*Suaeda salsa* L. in coastal saline-alkali areas	Egg yolk powder-induced hyperlipidemic zebrafish model	Hypolipidemic activity (100 μg/mL)	[179]
Fucoxanthin	Marine brown algae	Egg yolk powder-induced hyperlipidemic zebrafish model	Inhibits lipid accumulation (3.125, 6.25, 12.5 μM)	[180]
Glycosaminoglycans	*Ostrea rivularis*	Hyperlipidemic zebrafish	Hypolipidemic effect (fed with 125, 250, 500 mg/(kg·day))	[181]
Saringosterol acetate	*Sargassum fusiforme*	Diet-induced obese adult male zebrafish	Anti-obesity Activity (2.5% (*w*/*w*))	[182]
Extracts of cyanobacteria	cyanobacteria	Diet-induced obese zebrafish	Lipid Reducing Activity (10 μg/mL)	[168,169]
The Extracts of seagrass *Halophila stipulacea*	Seagrass *Halophila stipulacea*	Diet-induced obese zebrafish	Lipid Reducing Activity (2, 6 μg/mL)	[170]
fractions of cyanobacteria	cyanobacterial library	Diet-induced obese zebrafish	Repress intestinal lipid absorption (10 μg/mL)	[171]
Exometabolome from Cyanobacteria	Cyanobacteria	Diet-induced obese zebrafish	Lipid-Reducing Activity (25 µg/mL)	[172]
Chlorophyll derivative 13-2-hydroxypheophytine	Cyanobacteria	Diet-induced obese zebrafish	Reduce neutral lipid reserves (7.5 μg/mL)	[173]
Vitamin K1-analog (OH-PhQ)	Cyanobacterium *Tychonema* sp. LEGE 07196	Diet-induced obese zebrafish	Lipid reducing activity (10 μg/mL)	[174]
The Extracts of *Microbacterium foliorum* #91-29 and #91-40	*Microbacterium foliorum*	Diet-induced obese zebrafish	Lipid reducing activity (10 μg/mL)	[175]
Peptide LLTRAGL	*Rapana venosa*	2,4,6-trinitrobenzene sulfonic acid-induced *Tg(zlyz: EGFP)* zebrafish model	Protective effect against inflammatory bowel disease (20, 40, 80 μg/mL)	[190]
Septosone A	Marine Sponge *Dysidea* *septosa*	CuSO_4_-induced *Tg(zlyz: EGFP)* zebrafish model	Anti-inflammatory activity (2.5, 5, 10 μM)	[191]
Somalactam A	*Streptomyces somaliensis* 1107	LPS-induced *Tg(zlyz: EGFP)* zebrafish model	Anti-inflammatory effect (10 μM)	[192]
Dysidinoid B	Sponge *Dysidea septosa*	CuSO_4_-induced *Tg(zlyz: EGFP)* zebrafish model	Anti-inflammatory activity (20, 40, 80 μM)	[193]
Sarcoelegan A, (±)-sarcoelegan D, sarcoelegan E, (+)-sarcoelegan F, and (+)-sarcoelegan H	Soft coral *Sarcophyton elegans*	CuSO_4_-induced *Tg(zlyz: EGFP)* zebrafish	Anti-inflammatory activity (20 μM)	[112]
Altechromone A	Marine-derived fungus *Penicillium Chrysogenum* (XY-14-0-4)	CuSO_4_-, tail-cutting-, and LPS-induced *Tg(zlyz: EGFP)* zebrafish and TNBS-induced zebrafish model	Anti-inflammatory activity (12.5, 25, 50 μg/mL)	[194]
Purpurols A and B	Sponge *Pseudoceratina purpurea*	CuSO_4_-induced *Tg(zlyz: EGFP)* zebrafish	Anti-inflammatory activity (40 µM)	[195]
Protein hydrolysate AJH-1	sea cucumber *Apostichopus japonicus*	CuSO_4_-induced *Tg(zlyz: EGFP)* zebrafish	Anti-inflammatory activity (100, 250, 500 µg/mL)	[196]
Apo-9′-fucoxanthinone	*Sargassum muticum*	LPS-induced zebrafish model	Anti-inflammatory activity (25, 50, 100 μg/mL)	[197]
5-Hydroxypalisadin B	Red seaweed *Laurencia snackeyi*	LPS-induced and tail-cutting-zebrafish model	Anti-inflammatory activity (0.25, 0.5, 1 μg/mL)	[198]
Fucoidan (SFF-PS-F5)	Fermented *Sargassum fusiforme*	LPS-induced zebrafish model	Anti-inflammatory activity (25, 50, 100 μg/mL)	[199]
Fucoidan LJSF4	*Saccharina japonica*	LPS-induced zebrafish model	Anti-inflammatory effect (12.5–50 μg/mL)	[200]
Fucose containing sulfated polysaccharides (FCSP)	*Turbinaria ornata* from the Maldives	LPS-induced zebrafish model	Anti-inflammatory effect (12.5, 25, 50 μg/mL)	[201]
24-Methylcholesta-5(6), 22-Diene-3β-ol	Marine Diatom *Phaeodactylum tricornutum*	LPS-induced zebrafish model	Anti-inflammatory effect (12.5, 25, 50 μg/mL)	[202]
Sulfated polysaccharides (CFCE-PS)	Green seaweed *Codium fragile*	LPS-induced zebrafish model	Anti-inflammatory effect (25, 50, 100 μg/mL)	[203]
Sulfated polysaccharides (SFPS)	Brown Seaweed *Sargassum fulvellum*	LPS-induced zebrafish model	Anti-inflammatory effect (25, 50, 100 μg/mL)	[204]
Sulfated Galactofucan LJNF3	Brown seaweed *Saccharina japonica*	LPS-induced zebrafish model	Anti-inflammatory effect (12.5, 25, 50 μg/mL)	[205]
Eckmaxol and dieckol	Brown seaweed *Ecklonia maxima*	LPS-induced zebrafish model	Anti-inflammatory effect (12.5, 25, 50 μg/mL)	[206]
Enzymatic peptide SEP	Skipjack *Katsuwonus pelamis*	CuSO_4_-induced *Tg*(*zlyz: EGFP*) zebrafish	Anti-inflammatory activity (500 μg/mL)	[207]
Micrometam C	*Micromelum falcatum*	LPS-induced zebrafish model	Anti-inflammatory activity (10, 50, 200 μM)	[208]
Peptides YSQLENEFDR and YIAEDAER	Marine snail	metronidazole-treated *Tg (krt4: NTR-hKikGR)cy17* zebrafish	Antioxidant activity (0.77, 7.70, 76.98 and 1.03, 10.36, 103.63 μM)	[213]
Frondoplysin A	Marine sponge *Dysidea frondosa*	metronidazole-treated *Tg (krt4: NTR-hKikGR)cy17* zebrafish	Antioxidant activity (10, 20, 40 μM)	[214]
Peptide AFFP	Aquacultured flounder fish	2,2-azobis-(2-amidinopropane) hydrochloride-induced oxidative damage in a zebrafish model	Antioxidative effects (25, 50, 100 μg/mL)	[215]
Seahorse peptide SHP	*Hippocampus* *abdominalis*	AAPH-induced oxidative stress in the zebrafish model	Antioxidative effects (50, 100, 200 μg/mL)	[216]
Phloroglucinol, eckol, dieckol, eckstolonol and triphloroethol A	Brown algae *Ecklonia cava*	AAPH-induced oxidative stress in the zebrafish model	Antioxidative effects (50 μM)	[217]
Agaro-oligosaccharides AO	Agar of *Gracilaria lemaneiformis*	H_2_O_2_-stimulated oxidative stress in zebrafish	Antioxidant activity (12.5, 25, 50 μg/mL)	[218]
Xyloketal B	Mangrove fungus *Xylaria* sp. (no. 2508)	Phorbol myristate acetate-induced ROS levels in zebrafish	Antioxidant activity (20 μM)	[219]
(−)-loliolide	*Sargassum horneri*	AAPH-induced oxidative damage in zebrafish models	Antioxidant activity (6.25, 12.5, 25 μg/mL)	[220]
Protein hydrolysate HPH	Seahorses *Hippocampus abdominalis*	AAPH-induced oxidative damage in zebrafish	Antioxidant activity (100, 400 μg/mL)	[221]
Fucoidan (HFPS-F4)	*Hizikia fusiforme*	H_2_O_2_-stimulated oxidative stress in zebrafish	Antioxidant activity (12.5, 25, 50 μg/mL)	[222]

## 4. Conclusions

Marine natural products hold immense promise in drug discovery, necessitating animal models that simulate human biology and provide high-throughput, accurate screening for identifying bioactive lead molecules. Zebrafish, as a vertebrate model, bridges the gap between cellular and mammalian studies [8,11]. Compared to mammals, zebrafish offer advantages in reproduction, development, and feeding while maintaining high genetic and physiological conservation with humans. Zebrafish disease models allow for an effective exploration of disease mechanisms and solutions, making them valuable for activity screening. With advancements in automation and analysis technologies, zebrafish models enable high-throughput screening and economical evaluation of bioactive compounds with potential human benefits. They are widely used in biomedicine, personal care, functional foods, and natural product activity screening and evaluation [9,10].

This review summarized zebrafish models related to cardiovascular diseases, cancer, metabolic disorders, inflammation, and oxidative stress, emphasizing their use in evaluating marine natural products. Various zebrafish models have been developed, including chemically induced, diet-modified, and fluorescently labeled transgenic strains, which facilitate the observation of bioactive component effects. Among the research areas, our team has primarily focused on cardiovascular diseases, inflammation, and oxidative stress, though zebrafish models are also being increasingly applied in discovering marine bioactive ingredients, showcasing significant potential for future exploration. In addition to the zebrafish models in this review, there are some zebrafish models for neuroprotective activity, safety evaluation, and guidance on the isolation of active natural products [17]. The bioactive constituents obtained from terrestrial sources evaluated by zebrafish models have been applied in clinical research or marketed drugs, such as ferulic acid and danhong extracts. Sodium ferulate and danhong injection are extensively used to treat cardiovascular and cerebrovascular diseases in the clinic, and their proangiogenic activity was evaluated by a zebrafish model [223,224]. However, to our knowledge, there are currently few marine bioactive molecules evaluated by a zebrafish model in clinical research, indicating that zebrafish still have great potential in the exploration of marine bioactive molecules.

The application of the zebrafish model in studying marine bioactive molecules is still relatively limited compared to terrestrial sources, particularly traditional Chinese medicine, and requires more comprehensive and in-depth research. Many zebrafish models have not yet been fully applied to the discovery of marine bioactive molecules, and specific transgenic zebrafish models for certain diseases are still lacking. Therefore, further exploration and optimization of zebrafish models in this field are necessary. The zebrafish model also lacks standardized experimental operations, such as unified environmental conditions, technical procedures, and statistical analysis methods [17]. Establishing standardized experimental criteria and guidelines for each zebrafish model, along with building a database containing multiple models and their corresponding criteria, would greatly assist researchers in selecting appropriate models. Zebrafish models, constructed through gene manipulation techniques like specific gene knockout, overexpression or the introduction of target genes, are essential for studying the mechanisms of marine bioactive molecules and understanding their mechanistic relationships with human biology [8]. Although zebrafish genes are highly similar to those of humans, certain differences in the encoded proteins mean that drugs suitable for zebrafish may not achieve the same effects in humans. Therefore, confirming research results with other model organisms and clinical trials is crucial. Additionally, the larval zebrafish model poses challenges in quantifying drug administration, as substances are typically dissolved in water, making it difficult to measure true absorption and potentially leading to false negatives. More research is needed on bioavailability to overcome these limitations [17]. In conclusion, the potential of zebrafish models in screening marine bioactive molecules is significant, and with further advancements in science and technology, the discovery of marine bioactive molecules will make greater contributions to human medical research.

## Figures and Tables

**Table 1:** Comparison of commonly used animal models in drug discovery.

Organism	Nematode	Fruit fly	Zebrafish	Mouse
Generation time	3–4 days	11–12 days	3 months	2 months
Adult size	1–1.3 mm	3–4 mm	3–5 cm	6–10 cm
Embryos size	50 μm	100 μm	1–5 mm	N/A
Brood size	~140 eggs/day	~120 eggs/day	200–300 eggs/week	6–12 pups/month
Growth conditions	Solid or liquid medium	Solid medium	Liquid medium	Cages
Genome size	~97 Mb	~180 Mb	~1500 Mb	~3000 Mb
Homology to human (genome)	>50%	>60%	>80%	>90%
Transgenic organism Generation	weeks	weeks	months	months
Culture in microtiter plate	Eggs to adults	Eggs to larvae	Eggs to larvae	N/A
High-throughput drug screening	+++	++	++	N/A
Whole biological complexity	+	+	++	+++
Current use in drug discovery	+	+	++	+++
Ease of experimental operation	+++	++	++	+

+, ++, and +++, relative strength of the model in each category.

## Data Availability

No new data were generated.
